# Organ-Specific LC–MS/MS Phenolic Profiling and Multifunctional Antioxidant and Enzyme Inhibitory Activities of *Onosma sintenisii*

**DOI:** 10.3390/molecules31050840

**Published:** 2026-03-03

**Authors:** Zeyneb Karakus, Cengiz Sarikurkcu

**Affiliations:** 1 Chemical Technology Program, Çay Vocational School, Afyon Kocatepe University, 03200 Afyonkarahisar, Türkiye; zkarakus@aku.edu.tr; 2 Department of Analytical Chemistry, Faculty of Pharmacy, Afyonkarahisar Health Sciences University, 03100 Afyonkarahisar, Türkiye

**Keywords:** *Onosma sintenisii*, phenolic compounds, LC–MS/MS, hydroxycinnamic acids, antioxidant activity, enzyme inhibition, rosmarinic acid

## Abstract

*Onosma sintenisii* Hausskn. ex Bornm. is an endemic species of Türkiye whose phytochemical composition and biological activities remain insufficiently characterized at the organ level. The present study aimed to investigate organ-specific phenolic profiles and associated antioxidant and enzyme inhibitory activities of *O. sintenisii*. Ultrasonic-assisted methanolic extracts obtained from flowers, leaves, stems, and roots were analyzed using validated LC–ESI–MS/MS, and their biological potential was evaluated through multiple in vitro antioxidant assays (DPPH, ABTS, CUPRAC, FRAP, phosphomolybdenum, and metal chelation) as well as enzyme inhibition tests against acetylcholinesterase, butyrylcholinesterase, tyrosinase, α-amylase, and α-glucosidase. The results revealed pronounced organ-dependent variation in both phenolic composition and bioactivity. Rosmarinic acid was identified as the major phenolic compound in all organs, with the highest concentration detected in root extracts, which also exhibited the strongest antioxidant capacity and the most potent α-glucosidase inhibition. Flavonoid glycosides were predominantly accumulated in aerial parts. Correlation analysis demonstrated that hydroxycinnamic acids, particularly rosmarinic acid, are the main contributors to antioxidant and enzyme inhibitory effects. These findings indicate that *O. sintenisii*, especially its roots, represents a promising natural source of multifunctional phenolic compounds with potential pharmaceutical and nutraceutical applications.

## 1. Introduction

Medicinal and aromatic plants are widely recognized as natural sources of bioactive compounds, notably phenolic compounds and flavonoids, which contribute to antioxidant, anti-inflammatory, and enzyme inhibitory effects [1,2]. Increasing concerns about the side effects of synthetic agents have renewed interest in phytochemicals as safer alternatives in pharmaceutical and nutraceutical applications [3]. Among plant-derived metabolites, phenolics (especially phenolic acids and flavonoid glycosides) are of particular interest due to their multifunctional properties.

The genus *Onosma* L. (Boraginaceae) encompasses approximately 150 species distributed mainly across the Mediterranean Basin and Central Asia. Several *Onosma* taxa have been traditionally used in the treatment of burns, wounds, and inflammatory conditions, and their roots serve as a source of natural dyes [4,5]. Recent studies have confirmed that *Onosma* species are rich in phenolic acids (e.g., rosmarinic, caffeic, chlorogenic acids) and flavonoid derivatives (e.g., luteolin, apigenin glycosides), which underlie their strong antioxidant and enzyme inhibitory activities [6,7,8]. Notably, species such as *O. bourgaei*, *O. trachytricha*, and *O. mutabilis* have demonstrated significant enzyme inhibitory potential and LC–MS/MS-characterized phytochemical richness [6,9].

The genus *Onosma* L. (Boraginaceae) is characterized by distinctive morphological and anatomical features, including perennial or biennial growth habits, well-developed root systems, densely pubescent stems and leaves, and the frequent presence of trichomes and secretory structures that contribute to secondary metabolite accumulation [1,4]. Species of the genus are widely distributed across the Mediterranean Basin, Central Asia, and parts of the Middle East, where they occupy diverse ecological niches ranging from dry steppes and rocky slopes to gypsum-rich soils [1]. Ecologically, *Onosma* species play roles in plant–pollinator interactions and demonstrate adaptive strategies associated with environmental stress tolerance [5].

From an ethnobotanical perspective, several *Onosma* taxa have traditionally been used for wound healing, treatment of inflammatory conditions, and as natural dyes, particularly due to the pigment-rich roots [1,5]. These traditional uses have stimulated increasing scientific interest in the phytochemical diversity and biological activities of the genus [1]. In addition, the restricted distribution and habitat specificity of many *Onosma* species highlight their conservation relevance, emphasizing the importance of detailed phytochemical and biological investigations to support sustainable utilization and future pharmacological development [5].

Despite this growing body of literature, *Onosma sintenisii* Hausskn. ex Bornm., an endemic species in Türkiye, remains poorly explored from a phytochemical and biological perspective. Earlier studies have focused on its taxonomic and palynological features [10], while its chemical composition and bioactivities have only been addressed in a single LC–MS/MS-based study using whole aerial parts [11]. A detailed organ-specific analysis encompassing both phenolic composition and multiple bioactivity endpoints, including antioxidant and enzyme inhibitory assays, has not yet been reported.

Organ-specific metabolite distribution represents a fundamental aspect of plant secondary metabolism, reflecting tissue-dependent physiological roles and ecological adaptation. Phenolic compounds frequently accumulate differentially across plant organs, which may directly influence antioxidant mechanisms and enzyme inhibitory potential. Consequently, organ-resolved phytochemical profiling provides a more mechanistic framework for interpreting structure–activity relationships compared with whole-plant evaluations [12,13].

Accordingly, the present study aims to provide a comprehensive organ-specific phenolic characterization of *Onosma sintenisii* collected in Türkiye using LC–MS/MS and to relate these compositional differences to antioxidant performance and multi-target enzyme inhibition. By integrating phytochemical profiling with functional evaluation, this work seeks to clarify the biochemical basis underlying the biological potential of this species, thereby enabling a functional interpretation of organ-dependent bioactivity patterns.

## 2. Results

### 2.1. Extraction Yield, Total Phenolic and Flavonoid Contents

Ultrasonic-assisted methanolic extraction resulted in variable yields depending on the plant organ. The highest extraction yield was obtained from leaves (16.99%), followed by flowers (15.60%), stems (12.00%), and roots (3.36%).

**Figure 1:**
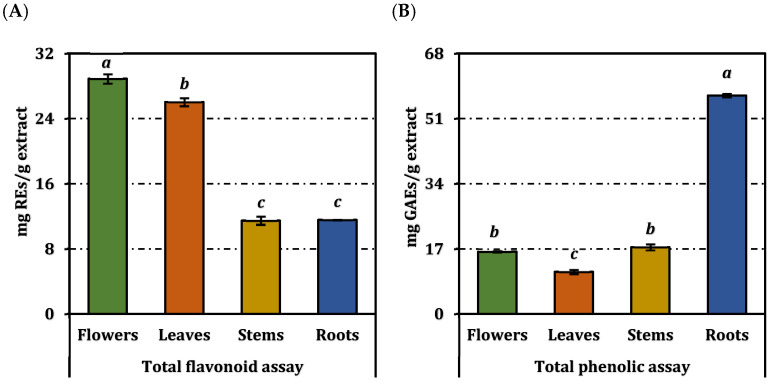
Total flavonoid and phenolic contents of *O. sintenisii* extracts. (**A**) Total phenolic content (TPC); (**B**) Total flavonoid content (TFC). REs and GAEs: Rutin and gallic acid equivalents, respectively. Values are expressed as mean ± standard deviation (n = 3). Bars indicated by different superscript letters are statistically significantly different according to one-way ANOVA followed by Tukey’s HSD post hoc test (*p* < 0.05).

As shown in Figure 1, the total phenolic content (TPC) of the extracts differed significantly among plant parts (*p* < 0.05).

The root extract exhibited the highest TPC value (57.03 ± 0.47 mg GAE/g extract), followed by stems (17.44 ± 0.80 mg GAE/g), flowers (16.28 ± 0.19 mg GAE/g), and leaves (10.99 ± 0.56 mg GAE/g). In contrast, total flavonoid content (TFC) followed a different trend. The highest TFC was recorded in flowers (28.90 ± 0.57 mg RE/g extract) and leaves (26.03 ± 0.50 mg RE/g), whereas stems (11.47 ± 0.52 mg RE/g) and roots (11.55 ± 0.04 mg RE/g) showed lower values

### 2.2. LC–MS/MS Analyses of Phenolic Compounds

LC–ESI–MS/MS analysis allowed for the identification and quantification of 16 phenolic compounds in the organ-specific methanolic extracts of *O. sintenisii* (Table 1).

Among these, rosmarinic acid was the predominant compound, most notably in the root extract (8039 ± 18 µg/g extract), followed by stems (4973 ± 14 µg/g), flowers (2047 ± 14 µg/g), and leaves (169 ± 4 µg/g). Chlorogenic acid was the second most abundant phenolic, especially in flowers (1527 ± 8 µg/g) and stems (1150 ± 5 µg/g). Caffeic acid was also prominent, with its highest content in stems (215 ± 1 µg/g).

Regarding flavonoids, luteolin-7-O-glucoside was detected at the highest levels in flowers (1116 ± 2 µg/g) and stems (589 ± 1 µg/g), while apigenin-7-O-glucoside showed a similar distribution. Flavonoid aglycones such as luteolin and apigenin were found in lower concentrations across all organs.

### 2.3. Antioxidant Activities

**Figure 2:**
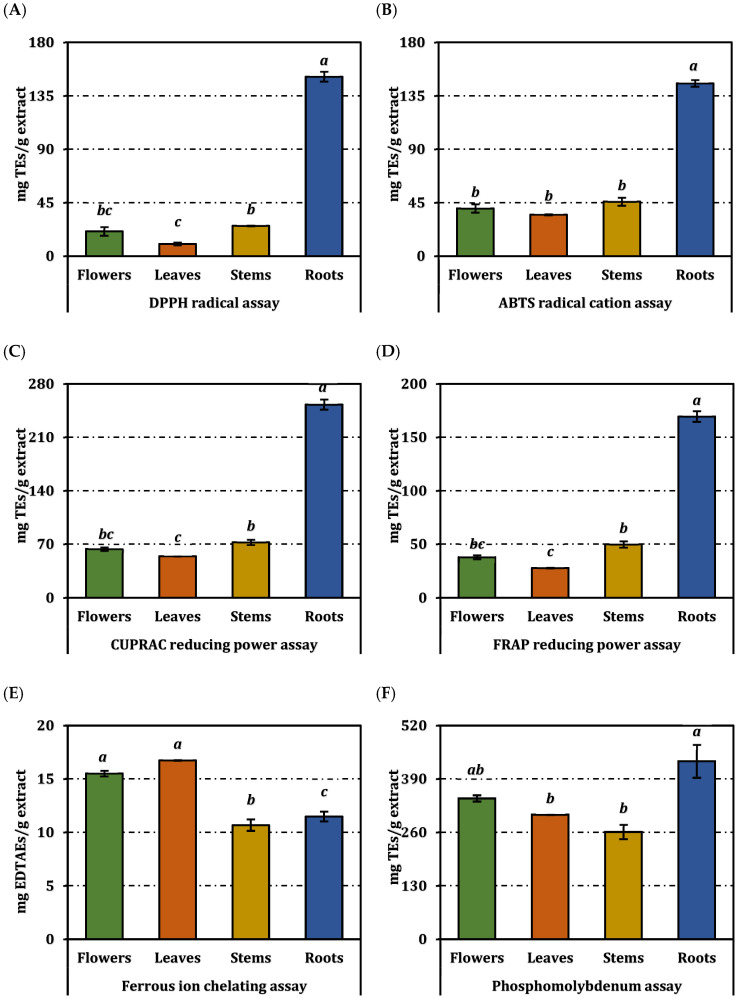
Antioxidant activity of *O. sintenisii* extracts. (**A**) DPPH; (**B**) ABTS; (**C**) CUPRAC; (**D**) FRAP; (**E**) Metal chelating; (**F**) Phosphomolybdenum activity. TEs and EDTAEs, trolox and ethylenediaminetetraacetic acid (disodium salt) equivalents, respectively. Values are expressed as mean ± standard deviation (n = 3). Bars indicated by different superscript letters are statistically significantly different according to one-way ANOVA followed by Tukey’s HSD post hoc test (*p* < 0.05).

The antioxidant potential of ultrasonic-assisted methanolic extracts obtained from the flowers, leaves, stems, and roots of *O. sintenisii* was evaluated using multiple in vitro assays based on different reaction mechanisms, including free radical scavenging, reducing power, and metal chelating activity. The antioxidant results were expressed as IC_50_/EC_50_ values and as standard equivalents; IC_50_/EC_50_ values are presented in Table 2, while the standard equivalent values are shown in Figure 2.

The root extract demonstrated the strongest antioxidant activity across all assays, with the lowest IC_50_ and EC_50_ values: DPPH (1.68 ± 0.05 mg/mL), ABTS (1.20 ± 0.02 mg/mL), CUPRAC (0.68 ± 0.02 mg/mL), FRAP (0.29 ± 0.01 mg/mL), and phosphomolybdenum (1.03 ± 0.10 mg/mL). In contrast, the flower and stem extracts exhibited weaker scavenging and reducing power. The equivalent-based results corroborated these findings, with the root extract displaying the highest Trolox equivalents for DPPH (151.17 ± 4.25 mg TE/g), ABTS (145.53 ± 2.88 mg TE/g), CUPRAC (252.88 ± 6.84 mg TE/g), and FRAP (169.46 ± 5.09 mg TE/g), while exhibiting moderate ferrous ion chelating activity (11.48 ± 0.46 mg EDTAE/g).

### 2.4. Enzyme Inhibitory Activity

**Figure 3:**
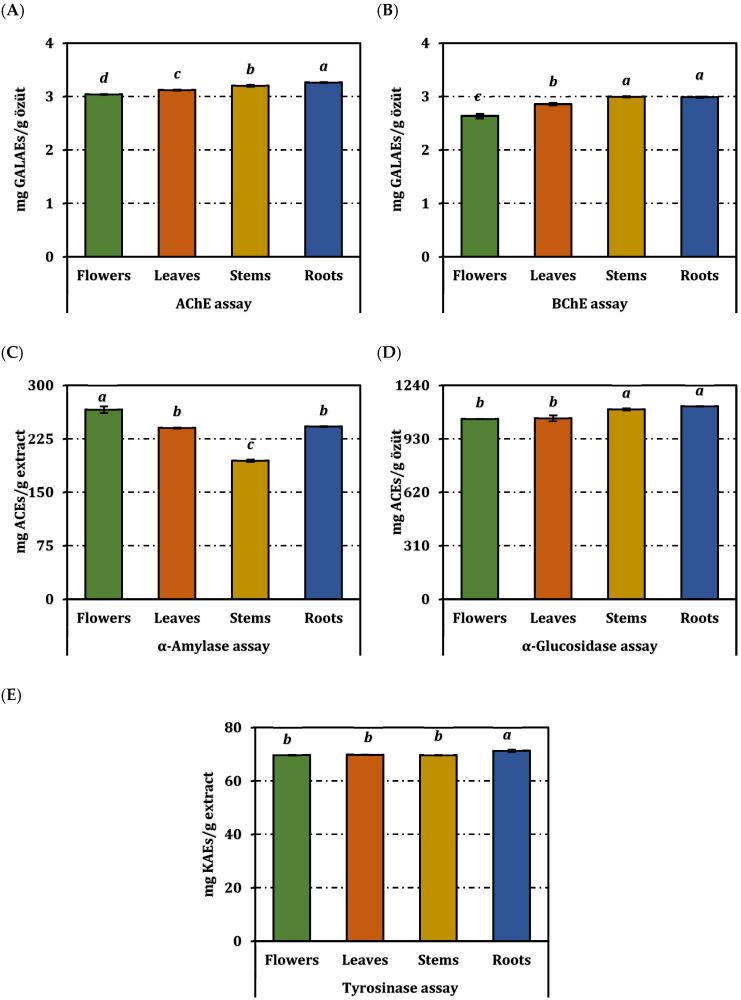
Enzyme inhibition activity of *O. sintenisii* extracts. (**A**) AChE inhibition; (**B**) BChE inhibition; (**C**) Tyrosinase inhibition; (**D**) α-amylase inhibition; (**E**) α-glucosidase inhibition. ACEs, GALAEs and KAEs mean acarbose, galanthamine and kojic acid equivalents, respectively. Values are expressed as mean ± standard deviation (n = 3). Bars indicated by different superscript letters are statistically significantly different according to one-way ANOVA followed by Tukey’s HSD post hoc test (*p* < 0.05).

The enzyme inhibitory potential of *O. sintenisii* extracts was assessed against five therapeutic targets: acetylcholinesterase (AChE) (from Electrophorus electricus, Sigma-Aldrich, St. Louis, MO, USA), butyrylcholinesterase (BChE) (from equine serum, Sigma-Aldrich, St. Louis, MO, USA), tyrosinase, α-amylase, and α-glucosidase. The results, expressed as IC_50_ values and standard equivalents, are presented in Table 3 and Figure 3.

Among the tested organs, the root extract exhibited the strongest AChE inhibitory activity with the lowest IC_50_ value (0.98 ± 0.003 mg/mL), which was statistically different from the other extracts (*p* < 0.05), while the flower extract showed weaker inhibition (IC_50_: 1.05 ± 0.003 mg/mL). For BChE, the root (1.07 ± 0.004 mg/mL) and stem extracts (1.07 ± 0.01 mg/mL) displayed statistically comparable inhibitory activity (*p* < 0.05), whereas the flower extract showed lower activity (1.21 ± 0.02 mg/mL).

Tyrosinase (from mushroom, Sigma-Aldrich, St. Louis, MO, USA) inhibition was moderate and comparable across organs, with IC_50_ values ranging from 1.16 to 1.18 mg/mL and kojic acid equivalents between 69.68 ± 0.11 and 71.33 ± 0.44 mg KAE/g.

The flower extract showed the strongest α-amylase (from porcine pancreas, Sigma-Aldrich, St. Louis, MO, USA) inhibitory activity (IC_50_: 3.53 ± 0.06 mg/mL; 266.14 ± 4.66 mg ACAE/g), whereas the root extract exhibited the strongest α-glucosidase (from Saccharomyces cerevisiae, Sigma-Aldrich, St. Louis, MO, USA) inhibition (IC_50_: 1.01 ± 0.001 mg/mL; 1119.53 ± 0.91 mg ACAE/g), with statistically significant differences among extracts (*p* < 0.05).

### 2.5. Correlation Analysis

**Figure 4:**
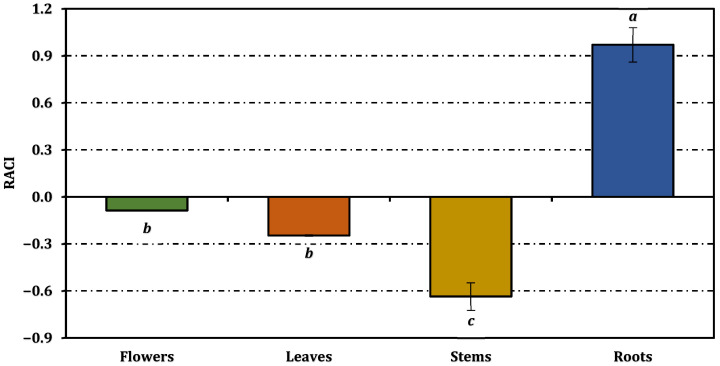
Relative antioxidant capacity index of *O. sintenisii* extracts. Values are expressed as mean ± standard deviation (n = 3). Bars indicated by different superscript letters are statistically significantly different according to one-way ANOVA followed by Tukey’s HSD post hoc test (*p* < 0.05).

**Figure 5:**
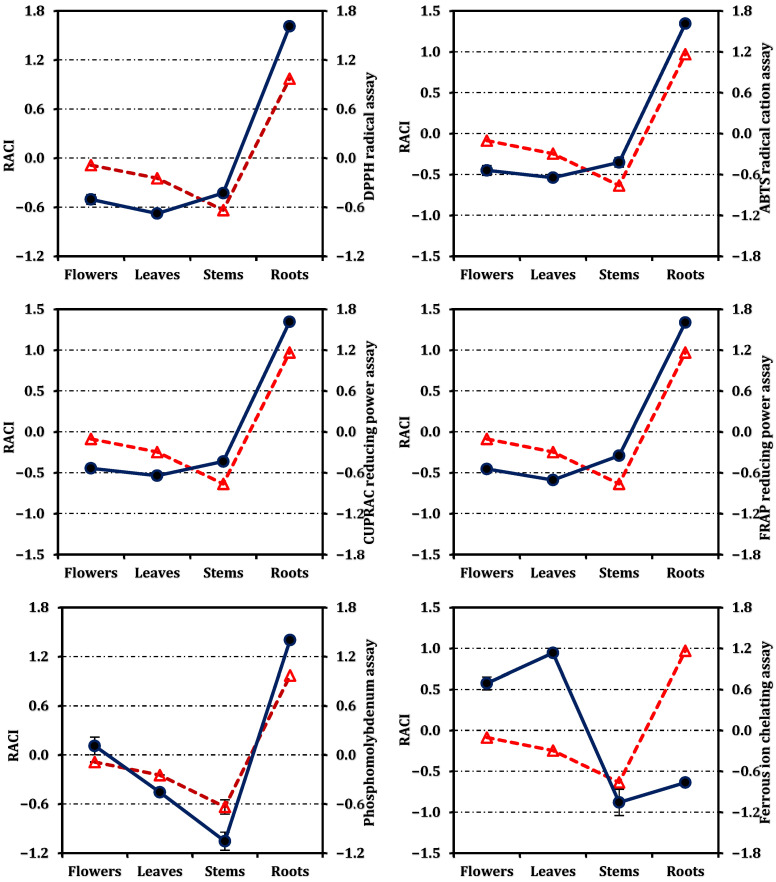
Correlation between the Relative antioxidant capacity index (dashed red line with triangle) and antioxidant activity (solid dark blue line with circle). Values are expressed as mean ± standard deviation (n = 3).

Based on the RACI values shown in Figure 4, the integrated antioxidant performance of *O. sintenisii* extracts followed a clear organ-dependent ranking, with roots (0.97) exhibiting the highest overall antioxidant capacity, followed by flowers (−0.09) and leaves (−0.25), whereas stems (−0.64) showed the lowest RACI value. In addition, the relationships between RACI values and individual antioxidant assays are illustrated in Figure 5. This ranking indicates that, when all antioxidant assays are considered collectively, the root extract provides the most consistent and strongest antioxidant performance across mechanisms, while the stem extract contributes the least.

Pearson correlation analysis was then used to explore linear relationships among antioxidant assays, enzyme inhibition tests, total phenolic/flavonoid contents, and individual phenolics quantified by LC–MS/MS (Table 4). Very strong positive correlations were observed among the electron transfer-based antioxidant assays (DPPH, ABTS, CUPRAC, FRAP) (e.g., DPPH–ABTS r = 0.998; DPPH–CUPRAC r = 0.999; ABTS–FRAP r = 0.997), indicating high internal consistency for these endpoints. In parallel, total phenolic content showed extremely strong positive correlations with these assays (r ≈ 0.998 with DPPH/ABTS/CUPRAC/FRAP), supporting a dominant contribution of phenolic constituents to radical scavenging and reducing power mechanisms.

In contrast, the ferrous ion chelating assay (FICA) displayed a markedly different behavior, showing moderate negative correlations with the electron transfer assays (FICA–DPPH r = −0.540; FICA–ABTS r = −0.539; FICA–FRAP r = −0.583).

## 3. Discussion

### 3.1. Extraction Yield, Total Phenolic and Flavonoid Contents

The extraction outcomes and global phenolic characteristics revealed a pronounced organ-dependent differentiation in *O. sintenisii*. Aerial organs were associated with higher extraction efficiency, whereas roots were distinguished by a markedly richer phenolic composition, indicating that extraction yield does not necessarily parallel phytochemical concentration. In contrast, flavonoid accumulation was more evident in flowers and leaves, pointing to a functional divergence between underground and photosynthetic tissues in terms of secondary metabolite allocation. This pattern suggests that differences in tissue structure and compound solubility play a key role in shaping organ-specific extraction behavior and phenolic distribution, and places the observed results within the broader context of medicinal plants in which phenolic acids preferentially accumulate in root tissues, as also reported for other *Onosma* species and root-rich taxa.

Organ-specific differences in extraction yield and phenolic composition observed in *O. sintenisii* are consistent with previous reports on *Onosma* species, where underground organs frequently accumulate higher levels of phenolic acids, while aerial parts tend to be richer in flavonoid glycosides [1,5]. This distribution likely reflects tissue-specific metabolic specialization and ecological adaptation described for Boraginaceae taxa [14]. In the present study, the elevated TPC and TFC values detected in root extracts were largely aligned with their superior antioxidant performance across multiple assays, as also supported by the RACI indices. However, minor variation among assays indicates that antioxidant activity is not solely determined by total phenolic concentration but is also influenced by compound composition and assay mechanism [14]. Overall, these findings suggest that organ-level phytochemical specialization plays a key role in shaping antioxidant capacity in *O. sintenisii*.

The observed differences between extraction yield and total phenolic content highlight the importance of distinguishing extraction efficiency from phytochemical concentration. Extraction yield primarily reflects the total amount of extractable compounds, including sugars, lipids, organic acids, and other non-phenolic constituents, whereas total phenolic content specifically represents the concentration of phenolic metabolites within the extract. Therefore, higher extraction yield does not necessarily correspond to higher phenolic richness, as demonstrated by the relatively low yield but high phenolic concentration observed in root extracts.

This phenomenon is closely linked to the physiological roles of secondary metabolites in plants. Roots often serve as storage and defense organs, accumulating phenolic acids as protective agents against soil-borne pathogens, oxidative stress, and environmental challenges [15]. Hydroxycinnamic acids, including rosmarinic acid and chlorogenic acid, are particularly known to accumulate in root tissues due to their antimicrobial and antioxidant functions [16].

The effectiveness of ultrasonic-assisted extraction employed in this study also contributes to enhanced phenolic recovery. Ultrasound facilitates cell wall disruption through cavitation effects, increasing solvent penetration and improving mass transfer, thereby enhancing the extraction of intracellular phenolic compounds. This technique has been shown to significantly improve extraction efficiency compared with conventional maceration methods, particularly for phenolic acids and flavonoids [17]

Collectively, these findings confirm that organ-specific metabolic specialization and optimized extraction techniques play a critical role in determining phenolic recovery and highlight the roots of *O. sintenisii* as a particularly rich source of bioactive phenolic compounds.

### 3.2. LC–MS/MS Analyses of Phenolic Compounds

LC–MS/MS analysis revealed that the phenolic composition of *O. sintenisii* differs markedly among plant organs. Rosmarinic acid was identified as the predominant constituent across all examined parts, whereas chlorogenic and caffeic acids were mainly associated with underground tissues. In contrast, aerial organs, particularly flowers and leaves, were characterized by a higher prevalence of flavone glycosides, including luteolin-7-O-glucoside and apigenin-7-O-glucoside. This organ-specific phenolic distribution allows a more systematic interpretation of previously reported data for *O. sintenisii* and facilitates comparison with phenolic profiles described for other *Onosma* species.

These results expand on a previous study by Ozer et al. [11], which reported the presence of rosmarinic acid and flavone glycosides in whole aerial parts of *O. sintenisii* without organ-level differentiation. Our findings confirm and extend this composition by showing that roots are particularly rich in phenolic acids, while aerial organs accumulate flavonoid glycosides.

When compared to other *Onosma* species, the rosmarinic acid content in *O. sintenisii* roots (8039 µg/g) exceeds that reported in *O. bourgaei* (5212 µg/g) and *O. mutabilis* (5743 µg/g) [6,9]. Similarly, luteolin derivatives were more abundant in *O. sintenisii* flowers than in those of *O. trachytricha*.

Overall, the LC–MS/MS data indicate a marked differentiation in the phenolic composition of *O. sintenisii* among plant organs, with hydroxycinnamic acids predominating in roots and flavone glycosides being more prevalent in aerial parts. The major constituents identified, including rosmarinic, chlorogenic, and caffeic acids as well as luteolin- and apigenin-based glycosides, provide a coherent phytochemical basis for the organ-dependent bioactivity patterns observed in the present study.

This organ-specific distribution likely reflects adaptive metabolic strategies, where phenolic acids accumulated in roots contribute to defense against soil-borne stressors and oxidative challenges, whereas the predominance of flavonoid glycosides in aerial tissues is commonly associated with photoprotection, UV screening, and responses to environmental stress. Such biochemical specialization has been widely described as a key mechanism underlying tissue-level functional differentiation in plants and supports the ecological and physiological roles of phenolic compounds in *Onosma* species [12,13].

From an application perspective, the observed organ-specific phenolic distribution provides important guidance for targeted extraction strategies. The high accumulation of hydroxycinnamic acids in roots suggests that root-derived extracts may represent efficient sources of natural antioxidants for nutraceutical and functional food applications, whereas aerial organs enriched in flavonoid glycosides may be more suitable for cosmetic and dermatological formulations due to their photoprotective and skin-related bioactivities. These findings therefore support the selective utilization of different plant organs depending on the intended industrial or pharmacological application [12,14].

These findings provide a strong biochemical foundation for understanding the antioxidant mechanisms discussed in the following section.

### 3.3. Antioxidant Activities

The antioxidant assessment of *O. sintenisii* extracts revealed a pronounced organ-dependent pattern across assays based on radical scavenging and reducing power. Among the examined plant parts, root extracts consistently exhibited the strongest antioxidant activity, whereas aerial organs displayed comparatively lower but still detectable effects. This antioxidant pattern indicates that the antioxidant capacity of *O. sintenisii* is largely governed by tissue-related biochemical characteristics.

These findings align with the study by Ozer et al. [11], which previously demonstrated the antioxidant activity of *O. sintenisii* methanolic extract using similar assays. However, the present study expands on their findings by revealing notable antioxidant activity, especially the higher activity of roots—a detail not addressed in the earlier whole-plant study.

Comparative analysis with other *Onosma* species further supports these results. For instance, *O. tauricum*, *O. ambigens*, and *O. riedliana* have shown antioxidant capacities comparable to or slightly lower than those observed in *O. sintenisii* root extracts, particularly in DPPH and CUPRAC assays [7,18,19]. The DPPH IC_50_ values for these species typically ranged from 1.5 to 3.0 mg/mL, while their CUPRAC equivalents ranged from 150 to 230 mg TE/g extract—values close to those obtained here for root extracts.

These comparisons confirm that the antioxidant capacity of *O. sintenisii* is within the expected range for the genus. From a comparative perspective, these findings position *O. sintenisii*, particularly its root extracts, among the more antioxidant-active members of the genus. The relatively high reducing capacity and consistent performance across multiple assays suggest that this species represents a phenolic-acid-dominant antioxidant model within *Onosma*. This positioning indicates that the antioxidant efficiency of *O. sintenisii* is not merely comparable but mechanistically coherent with its phytochemical profile, reinforcing its relevance as a candidate species for natural antioxidant development and future pharmacological exploration [1,14]. These observations are consistent with the predominance of hydroxycinnamic acid derivatives, particularly rosmarinic acid, which are known to contribute strongly to electron-transfer-based antioxidant responses and reducing capacity across multiple assay systems.

From a mechanistic perspective, the antioxidant capacity of *O. sintenisii* is closely linked to its dominant phenolic constituents identified via LC–MS/MS, particularly rosmarinic acid, chlorogenic acid, caffeic acid, luteolin-7-O-glucoside, and apigenin-7-O-glucoside. These compounds are known to exert their antioxidant effects through electron transfer, hydrogen atom donation, and metal ion chelation mechanisms. Rosmarinic acid, which accumulated predominantly in root extracts, has been consistently associated with strong free-radical scavenging and reducing power in related *Onosma* species such as *O. riedliana*, *O. tauricum*, and *O. bulbotrichum* [7,18,20]. Similarly, chlorogenic and caffeic acids, abundant in both root and stem extracts, are widely reported for their role in electron transfer and radical stabilization [19,21]. Luteolin-7-O-glucoside and apigenin-7-O-glucoside, detected at high levels in flowers and leaves, contribute to metal chelating activity and secondary antioxidant effects, as demonstrated in studies on *O. mitis* and *O. ambigens* [19,22]. While glycosylation may slightly reduce their direct radical scavenging capacity, it enhances solubility and bioavailability, sustaining their antioxidant function over time [14,23]. These organ-specific patterns reflect conserved structure–function relationships observed across the *Onosma* genus.

Phenolic antioxidants act mainly through electron transfer, hydrogen donation, and metal chelation mechanisms, which are strongly influenced by structural features such as hydroxyl group arrangement and conjugation [24].

Rosmarinic acid, the major phenolic constituent identified in this study, possesses two catechol moieties, which significantly enhance its electron-donating capacity and radical scavenging efficiency. This structural configuration enables rosmarinic acid to function as a highly effective chain-breaking antioxidant and metal chelator. Previous studies have demonstrated that rosmarinic acid exhibits antioxidant activity comparable to or exceeding that of conventional antioxidants such as Trolox and ascorbic acid, confirming its role as a key contributor to the antioxidant capacity of phenolic-rich plant extracts [16].

The strong performance of root extracts in reducing power assays such as FRAP and CUPRAC further supports the predominance of electron transfer-based antioxidant mechanisms. These assays specifically measure the ability of antioxidants to donate electrons and reduce metal ions, reflecting the reducing potential of phenolic compounds. The high reducing capacity observed in root extracts therefore reflects their elevated concentration of redox-active hydroxycinnamic acids, particularly rosmarinic acid and chlorogenic acid [25].

In addition to radical scavenging and reducing mechanisms, phenolic compounds also contribute to antioxidant defense through metal ion chelation, thereby preventing the formation of highly reactive hydroxyl radicals via Fenton-type reactions. This multifunctional antioxidant behavior enhances the overall protective potential of phenolic-rich plant extracts and underscores the pharmacological relevance of *O. sintenisii* as a natural antioxidant source [14].

Collectively, these findings demonstrate that the antioxidant capacity of *O. sintenisii* is primarily driven by its phenolic composition, particularly hydroxycinnamic acids, and confirm the roots of this species as a highly promising source of natural antioxidants with potential applications in pharmaceutical, nutraceutical, and functional food development.

### 3.4. Enzyme Inhibitory Activity

Evaluation of the enzyme inhibitory properties of *O. sintenisii* extracts demonstrated a differentiated inhibition profile among the examined plant parts across all tested enzymatic targets. Distinct differences were observed among plant parts, with aerial organs and roots contributing differentially to the inhibition of cholinesterases, tyrosinase, and carbohydrate-hydrolyzing enzymes. This heterogeneous inhibition pattern indicates that enzyme inhibitory activity in *O. sintenisii* is closely associated with organ-specific phytochemical composition rather than reflecting a uniform inhibitory potential across the plant. The observed organ-level differentiation provides a coherent basis for contextualizing the present findings with previously reported data on *O. sintenisii* and for comparison with enzyme inhibition patterns described for other *Onosma* species.

These findings are consistent with Ozer et al. [11], who first reported enzyme inhibitory activity of *O. sintenisii* methanolic extracts, though without organ-specific differentiation. Our organ-based results further refine this understanding by demonstrating that flower and root extracts are the main contributors to cholinesterase and glycosidase inhibition, respectively [11]. These findings are also comparable to other *Onosma* species reported in the literature. For instance, *O. bourgaei*, *O. trachytricha*, *O. mollis*, and *O. ambigens* exhibited inhibitory activity against AChE, BChE, tyrosinase, α-amylase, and α-glucosidase, though specific potency varied depending on plant part and extraction method [6,19,26]. Similarly, *O. heterophyllum* demonstrated broad-spectrum enzyme inhibition, reinforcing the notion that polyphenol-mediated enzyme inhibition is a consistent pharmacological feature across the genus *Onosma*.

From an interpretive perspective, the enzyme inhibition profile observed for *O. sintenisii* indicates that this species may represent a comparatively strong multifunctional inhibitor within the genus, particularly regarding cholinesterase and glycosidase targets. The pronounced AChE and α-glucosidase inhibition shown by root extracts suggests potential neuroprotective and antidiabetic relevance, while the consistent tyrosinase inhibition across organs supports possible dermatological applications. These findings therefore extend the significance of the results beyond descriptive activity reporting and highlight the pharmacological positioning of *O. sintenisii* as a promising source of enzyme-modulating phenolic compounds [14].

The observed enzyme inhibition can be mechanistically linked to the phenolic composition of the extracts. Rosmarinic acid, chlorogenic acid, caffeic acid, luteolin-7-O-glucoside, and apigenin-7-O-glucoside—all identified in *O. sintenisii*—have demonstrated notable enzyme inhibitory activities in previous studies. Rosmarinic acid, in particular, exhibits strong binding to both AChE and BChE by engaging catalytic and peripheral sites through π–π stacking and hydrogen bonding [27,28]. Chlorogenic acid also inhibits cholinesterases competitively, while caffeic acid has been shown to lower brain cholinesterase activity in vivo [29,30].

Luteolin-7-O-glucoside and apigenin-7-O-glucoside contribute to AChE inhibition as well, with luteolin glycoside exhibiting notably higher activity due to its catechol substitution, enhancing active site interactions [31]. Docking studies confirm these flavone glycosides can form stable complexes with AChE and BChE [6].

Rosmarinic and chlorogenic acids also contribute significantly to tyrosinase inhibition, likely through copper chelation and binding at the active site [32,33,34]. While caffeic acid alone shows limited tyrosinase inhibitory effect, its esterified derivative chlorogenic acid exhibits stronger inhibition, likely due to altered binding orientation and additional interactions within the enzyme’s active site [35].

Regarding glycosidase inhibition, caffeic acid has been reported as particularly effective, with IC_50_ values in the low micromolar range. Chlorogenic acid, though slightly less potent, still inhibits both α-amylase and α-glucosidase effectively. Rosmarinic acid and luteolin-7-O-glucoside also contribute, with molecular docking and in vitro studies supporting their inhibitory effects [6,29].

Taken together, these inhibition patterns indicate that multiple phenolic subclasses contribute differentially to enzyme modulation in *O. sintenisii*. Their presence provides a strong biochemical rationale for the potential use of this species in neuroprotective, antidiabetic, and dermatological applications.

The enzyme inhibitory activity observed in *O. sintenisii* extracts has important pharmacological implications, as the targeted enzymes are directly associated with major human diseases. Cholinesterase inhibitors are widely used in the management of Alzheimer’s disease to enhance cholinergic neurotransmission, while α-glucosidase and α-amylase inhibitors play a critical role in controlling postprandial hyperglycemia in patients with type 2 diabetes mellitus. Similarly, tyrosinase inhibitors are of significant interest in dermatological applications for the treatment of hyperpigmentation disorders and in cosmetic formulations [36].

The enzyme inhibitory activity observed in this study is consistent with the phenolic profile identified by LC–MS/MS, where hydroxycinnamic acids and flavonoid glycosides are known to interact with enzyme active sites through hydrogen bonding and π–π interactions, supporting the mechanistic basis of the observed inhibition [14,27].

Rosmarinic acid, which was detected at high concentrations in root extracts, has been shown to exhibit strong inhibitory effects against cholinesterases and carbohydrate-hydrolyzing enzymes. Its inhibitory activity is largely attributed to its ability to interact with both catalytic and peripheral binding sites of enzymes, thereby reducing substrate access and enzyme turnover. Similarly, chlorogenic acid and caffeic acid contribute to enzyme inhibition through competitive and non-competitive mechanisms, further enhancing the overall inhibitory potential of phenolic-rich extracts [37].

Flavonoid glycosides such as luteolin-7-O-glucoside and apigenin-7-O-glucoside also play a significant role in enzyme inhibition. Their structural features, including hydroxyl group arrangement and conjugated aromatic systems, facilitate interaction with enzyme active sites and contribute to inhibitory activity. Although glycosylation may influence binding affinity, these compounds retain significant inhibitory potential and contribute synergistically to the overall activity of the extracts [14,23].

Collectively, the enzyme inhibition profile observed in *O. sintenisii* extracts reflects the combined action of multiple phenolic compounds and highlights the pharmacological potential of this species as a natural source of enzyme inhibitors with possible applications in the prevention and management of neurodegenerative, metabolic, and dermatological disorders.

### 3.5. Correlation Analysis

To further clarify the relationships between phytochemical composition and biological activities, correlation analysis was performed. Correlation analysis revealed distinct association patterns among antioxidant assays, enzyme inhibitory activities, total phenolic and flavonoid contents, and individual phenolic constituents, indicating that different biochemical mechanisms contribute selectively to the observed bioactivities. Strong concordance was observed among assays based on reducing power and radical scavenging, whereas metal chelating activity exhibited a contrasting relationship with these endpoints, reflecting a separation between electron transfer-driven antioxidant behavior and chelation-based mechanisms. This differentiated correlation structure suggests that phenolic subclasses do not contribute uniformly across antioxidant systems but rather exhibit mechanism-dependent activity profiles, thereby providing an appropriate framework for interpreting the mechanistic divergence between electron transfer-based assays and metal chelation described below.

This divergence is mechanistically expected because metal chelation reflects ligand–metal complex formation rather than direct electron transfer (ET)/hydrogen donation; therefore, compounds driving ET-based assays do not necessarily drive chelating capacity, and inverse patterns across organs may occur when different phenolic subclasses dominate different mechanisms [14,25].

Importantly, the correlations of the major LC–MS/MS constituents help explain this split in antioxidant behavior. Rosmarinic acid showed strong positive correlations with the ET-based assays (DPPH r = 0.867; ABTS r = 0.864; CUPRAC r = 0.861; FRAP r = 0.888) and a strong negative correlation with FICA (r = −0.879), consistent with its association with electron transfer/reducing behavior rather than chelation under these conditions.

In contrast, chlorogenic acid exhibited negative correlations with ET-based assays (DPPH r = −0.715; ABTS r = −0.724; CUPRAC r = −0.720; FRAP r = −0.747) but a strong positive correlation with FICA (r = 0.864). Similarly, the dominant flavonoid glycosides luteolin-7-glucoside and apigenin-7-glucoside showed negative correlations with ET-based assays (e.g., luteolin-7-glucoside: DPPH r = −0.689; ABTS r = −0.706; FRAP r = −0.701; apigenin-7-glucoside: DPPH r = −0.473; ABTS r = −0.492; FRAP r = −0.501) while correlating positively with FICA (r = 0.452 and r = 0.583, respectively).

Although these compounds are quantitatively “major”, their negative correlations with ET-based antioxidant assays do not imply a lack of antioxidant relevance; rather, they likely reflect (i) mechanism-dependence of assays (ET vs. chelation) and (ii) the organ-level covariance structure of the dataset, where root extracts simultaneously show very high ET-based antioxidant responses and very low concentrations of chlorogenic acid and flavonoid glycosides relative to flowers/leaves. In addition, glycosylation is widely reported to modulate antioxidant behavior by affecting hydroxyl availability and redox properties compared with aglycones, leading to assay-dependent performance differences for flavonoid glycosides [14,23].

Finally, Pearson analysis also indicated meaningful associations between antioxidant endpoints and enzyme inhibition. For example, tyrosinase inhibition correlated strongly with ET-based antioxidant assays (TIA–DPPH r = 0.951; TIA–FRAP r = 0.954), whereas α-amylase inhibition showed weak correlations with ET-based assays (e.g., AAIA–DPPH r = 0.102) but a positive correlation with FICA (r = 0.665), suggesting that different chemical features may underlie inhibition patterns depending on the enzyme system.

When the Pearson correlation patterns obtained in the present study are evaluated in the context of previous reports on *Onosma* species, a high degree of interspecific consistency becomes evident. Strong positive correlations between total phenolic content and electron transfer-based antioxidant assays (DPPH, ABTS, CUPRAC, FRAP) have been widely reported across the genus, confirming phenolics as the primary drivers of reducing power and radical scavenging capacity. Conversely, metal chelating activity has repeatedly shown weak or negative associations with ET-based assays, reflecting a mechanistic distinction between ligand–metal complex formation and direct electron or hydrogen donation. At the level of individual compounds, rosmarinic acid consistently correlates with ET-based antioxidant responses, whereas chlorogenic acid and dominant flavonoid glycosides are more closely associated with chelation-related behavior. The correlation structure observed for *O. sintenisii* therefore aligns with a conserved, mechanism-dependent antioxidant pattern previously described for the genus *Onosma*.

Finally, the relationship between antioxidant capacity and enzyme inhibition observed in *O. sintenisii* also parallels earlier *Onosma* studies. In *O. riedliana* and *O. ambigens*, tyrosinase inhibition was reported to correlate more strongly with ET-based antioxidant assays than with metal chelation, whereas α-amylase inhibition showed weaker or inconsistent associations with reducing power but occasional links to chelating capacity [18,19]. The correlation profile observed in Table 4—namely, strong ET-based associations for tyrosinase inhibition and a preferential link between α-amylase inhibition and FICA—thus fits well within the broader enzymatic inhibition landscape previously described for the genus.

While correlation analysis provides valuable insight into potential structure–activity relationships, it is important to note that correlation does not necessarily imply direct causation. The observed associations reflect statistical relationships that may arise from the co-occurrence of multiple bioactive compounds within the extracts rather than the exclusive contribution of a single compound. Nevertheless, the strong correlations observed between total phenolic content, individual hydroxycinnamic acids, and antioxidant activity strongly support the central role of phenolic compounds as primary determinants of antioxidant capacity.

The differential correlation patterns observed between electron transfer-based assays and metal chelating activity further confirm that distinct subclasses of phenolic compounds contribute preferentially to different antioxidant mechanisms. Hydroxycinnamic acids, particularly rosmarinic acid, exhibit strong reducing power and radical scavenging activity due to their favorable redox potential, whereas flavonoid glycosides may contribute more significantly to metal chelation and secondary antioxidant mechanisms. These findings align with well-established structure–activity relationships reported for phenolic compounds, in which antioxidant activity is closely linked to hydroxyl group arrangement, conjugation, and molecular structure [14,24].

Importantly, the correlation between antioxidant capacity and enzyme inhibitory activity observed in this study further highlights the multifunctional pharmacological potential of phenolic compounds. Phenolic antioxidants can modulate enzyme activity not only through direct binding to enzyme active sites but also by influencing the oxidative environment, which may alter enzyme conformation and catalytic efficiency [14,38]. In addition, phenolic compounds have been shown to interact with enzymes through hydrogen bonding, hydrophobic interactions, and metal ion chelation, thereby contributing to enzyme inhibition and biological regulation [36]. This multifunctional behavior enhances the therapeutic relevance of phenolic-rich plant extracts and supports the potential application of *O. sintenisii* as a source of bioactive compounds for pharmaceutical and nutraceutical development [39].

Collectively, the correlation analysis provides strong mechanistic support for the role of phenolic compounds as key drivers of both antioxidant and enzyme inhibitory activities and confirms the biochemical and pharmacological significance of the phenolic profile identified in *O. sintenisii*.

## 4. Materials and Methods

### 4.1. Plant Material

*Onosma sintenisii* Hausskn. ex Bornm. was collected at the full flowering stage on 1 August 2025 from gypsum-rich regions of Kümbet village (Zara district, Sivas Province, Türkiye; 39°47′13″ N, 37°49′16″ E; 1920 m a.s.l.). Taxonomic identification was confirmed by Dr. Bedrettin Selvi, and a voucher specimen (GOPU 9601) was deposited at the Herbarium of the Faculty of Arts and Sciences, Tokat Gaziosmanpaşa University. Flowers, leaves, stems, and roots were separated, shade-dried at room temperature, ground into powder, and stored in airtight containers until extraction.

### 4.2. Extraction Procedure

Ultrasonic-assisted extraction was employed to enhance phenolic recovery from individual organs [17]. For each plant part, 5 g of dried powder was extracted with 100 mL methanol (Merck, Darmstadt, Germany) in an ultrasonic bath (Bandelin Sonorex RK100H, Berlin, Germany) at 30 °C for 1 h. Extracts were filtered and evaporated (Buchi R-210, Flawil, Switzerland) under reduced pressure, and the dry residues were stored at 4 °C until analysis.

### 4.3. Determination of Total Phenolic and Flavonoid Content

Total phenolic content (TPC) was determined using the Folin–Ciocalteu (Sigma-Aldrich, St. Louis, MO, USA) method and expressed as mg gallic acid (Sigma-Aldrich, St. Louis, MO, USA) equivalents per gram extract (mg GAE/g extract) [40]. Total flavonoid content (TFC) was measured by the aluminum chloride (Merck, Darmstadt, Germany) colorimetric method and expressed as mg rutin (Sigma-Aldrich, St. Louis, MO, USA) equivalents per gram extract (mg RE/g extract) [41]. Detailed protocols are provided in the Supplementary Materials.

### 4.4. LC–ESI–MS/MS Analysis

Individual phenolic compounds were quantified using liquid chromatography–electrospray ionization tandem mass spectrometry (LC–ESI–MS/MS), based on a validated method [42]. An Agilent 1260 Infinity LC system (Agilent Technologies, Santa Clara, CA, USA) coupled to a 6420 Triple Quadrupole MS was used with a Poroshell 120 EC-C18 column (100 mm × 4.6 mm, 2.7 µm; Agilent Technologies, Santa Clara, CA, USA). The mobile phase consisted of 0.1% formic acid (A) and methanol (B), applied in a gradient mode at a flow rate of 0.4 mL/min. Quantification was achieved by multiple reaction monitoring (MRM), comparing retention times and transitions with authentic standards. Instrumental parameters, chromatographic conditions, and compound-specific data are summarized here, while the complete operational settings are provided in the Supplementary Materials (Tables S1 and S2).

### 4.5. Antioxidant Activity Assays

Antioxidant capacity was evaluated via six in vitro assays: DPPH (Sigma-Aldrich, St. Louis, MO, USA) [43], ABTS (Sigma-Aldrich, St. Louis, MO, USA) [44], CUPRAC [25], FRAP [45], metal chelating activity [46], and phosphomolybdenum assay [47]. Results were expressed as IC_50_ or EC_50_ values and standard equivalents [e.g., Trolox (Sigma-Aldrich, St. Louis, MO, USA), EDTA]. Detailed assay conditions are described in the Supplementary Materials.

### 4.6. Enzyme Inhibition Assays

Enzyme inhibitory activities against acetylcholinesterase (AChE), butyrylcholinesterase (BChE), tyrosinase, α-amylase, and α-glucosidase were evaluated using microplate-based spectrophotometric assays (BioTek Synergy HTX, Winooski, VT, USA). Methanolic extracts were prepared at 10 mg/mL and serially diluted to obtain working concentrations.

Cholinesterase inhibition was determined using Ellman’s method [48]. Sample solutions were mixed with enzyme solution and DTNB reagent (Sigma-Aldrich, St. Louis, MO, USA) in Tris–HCl buffer (pH 8.0) and pre-incubated at 25 °C for 15 min. The reaction was initiated by adding acetylthiocholine iodide (Sigma-Aldrich, St. Louis, MO, USA) or butyrylthiocholine chloride (Sigma-Aldrich, St. Louis, MO, USA) as substrates, and absorbance was measured at 405 nm.

Tyrosinase inhibitory activity was evaluated using L-DOPA (Sigma-Aldrich, St. Louis, MO, USA) as substrate [49]. Sample solution was combined with enzyme solution and phosphate buffer (pH 6.8), pre-incubated, and the reaction was initiated with L-DOPA. Absorbance was recorded at 492 nm.

α-Amylase inhibition was assessed using the iodine/potassium iodide method [50]. Sample solutions were pre-incubated with enzyme solution, followed by addition of starch substrate. The reaction was terminated with HCl, iodine reagent was added, and absorbance was measured at 630 nm.

α-Glucosidase inhibitory activity was determined using PNPG (Sigma-Aldrich, St. Louis, MO, USA) as substrate [51]. Sample solutions were mixed with enzyme solution in phosphate buffer, incubated, and the reaction was stopped with sodium carbonate before measuring absorbance at 400 nm.

IC_50_ values were calculated from dose–response curves. Galanthamine, kojic acid, and acarbose were used as reference inhibitors for cholinesterase, tyrosinase, and glycosidase assays, respectively. Enzyme inhibitory activities were expressed both as IC_50_ values and as standard equivalents relative to the corresponding reference compounds. Detailed experimental conditions are provided in the Supplementary Materials.

### 4.7. Relative Antioxidant Capacity Index (RACI)

RACI values were calculated by standardizing results from individual antioxidant assays to enable integrated comparison, as described by Sun and Tanumihardjo [52] and Petrovic et al. [53]. The full calculation approach is provided in the Supplementary Materials.

### 4.8. Statistical Analysis

All experiments were performed in triplicate using independent replicates, and results are reported as mean ± standard deviation. Data normality was verified using the Shapiro–Wilk test prior to statistical comparisons. Homogeneity of variances was assessed using Levene’s test. Statistical differences among groups were evaluated by one-way ANOVA followed by Tukey’s HSD post hoc test using IBM SPSS v22.0. Statistical significance was accepted at *p* < 0.05.

### 4.9. Use of Artificial Intelligence

The authors declare that artificial intelligence-assisted tools were used solely for language editing and grammar checking to improve the clarity and readability of the manuscript. No artificial intelligence tool was used for data generation, data analysis, interpretation of results, or scientific decision-making. All scientific content, interpretations, and conclusions were generated by the authors, who take full responsibility for the integrity and originality of the work.

## 5. Conclusions

This study provides a comprehensive organ-specific characterization of the phenolic composition and associated biological activities of *O. sintenisii*, an endemic species of Türkiye, using validated LC–ESI–MS/MS analysis in combination with multiple antioxidant and enzyme inhibition assays. The results clearly demonstrate pronounced organ-dependent variation in both phytochemical composition and biological activity.

Hydroxycinnamic acids, particularly rosmarinic acid, were identified as the dominant phenolic constituents, with exceptionally high concentrations detected in root extracts. This compound, together with chlorogenic acid, caffeic acid, and flavonoid glycosides, provides a strong biochemical basis for the antioxidant and enzyme inhibitory activities observed. The superior antioxidant capacity and enzyme inhibitory potential of root extracts highlight the importance of organ-specific evaluation in identifying pharmacologically relevant plant materials.

Correlation analysis further confirmed that phenolic compounds represent the primary drivers of biological activity, revealing distinct structure–activity relationships and mechanistic differences between antioxidant pathways. These findings demonstrate that hydroxycinnamic acids are primarily responsible for electron transfer-based antioxidant activity, whereas flavonoid glycosides may contribute to complementary antioxidant and enzyme inhibitory mechanisms.

Importantly, the multifunctional bioactivity profile observed in *O. sintenisii*, including antioxidant, cholinesterase inhibitory, tyrosinase inhibitory, and glycosidase inhibitory effects, underscores its potential as a valuable natural source of bioactive compounds. These biological properties suggest possible applications in the prevention and management of oxidative stress-related disorders, neurodegenerative diseases, metabolic disorders, and dermatological conditions.

From a phytochemical and pharmacological perspective, the present study significantly advances current knowledge of the genus *Onosma* and provides new insights into the organ-specific distribution of bioactive phenolic compounds. The use of LC–MS/MS-based profiling combined with integrated bioactivity assessment offers a robust framework for identifying biologically relevant plant-derived compounds.

Although further in vivo and clinical studies are required to fully establish therapeutic efficacy and safety, the findings presented here strongly support the potential of *O. sintenisii* as a promising natural source of multifunctional phenolic compounds with potential applications in pharmaceutical, nutraceutical, and functional food development.

## Figures and Tables

**Table 1:** Concentration (µg/g extract) of selected phenolic compounds in *O. sintenisii* extracts.

No	Compounds	Flowers	Leaves	Stems	Roots
1	Rosmarinic acid	2047 ± 14 *^c^*	169 ± 4 *^d^*	4973 ± 14 *^b^*	8039 ± 18 *^a^*
2	Chlorogenic acid	1527 ± 8 *^a^*	1150 ± 5 *^b^*	490 ± 3 *^c^*	245 ± 4 *^d^*
3	Luteolin-7-O-glucoside	1116 ± 2 *^a^*	491 ± 1 *^c^*	589 ± 1 *^b^*	168 ± 5 *^d^*
4	Apigenin-7-O-glucoside	696 ± 1 *^a^*	182 ± 13 *^b^*	82.7 ± 0.7 *^c^*	25.0 ± 0.4 *^d^*
5	Caffeic acid	145 ± 4 *^c^*	44.0 ± 0.1 *^d^*	215 ± 1 *^a^*	159 ± 1 *^b^*
6	4-Hydroxybenzoic acid	88.9 ± 2.2 *^c^*	324 ± 2 *^a^*	82.1 ± 0.5 *^c^*	99.3 ± 1.3 *^b^*
7	3-Hydroxybenzoic acid	86.7 ± 1.8 *^c^*	317 ± 3 *^a^*	81.0 ± 0.9 *^c^*	102 ± 6 *^b^*
8	Verbascoside	67.7 ± 2.5 *^b^*	35.8 ± 0.6 *^c^*	65.4 ± 1.6 *^b^*	138 ± 2 *^a^*
9	Apigenin	51.6 ± 1.1 *^a^*	20.6 ± 0.1 *^b^*	7.44 ± 0.15 *^c^*	3.45 ± 0.04 *^d^*
10	Hesperidin	47.3 ± 0.6 *^a^*	7.97 ± 0.19 *^d^*	26.9 ± 0.1 *^b^*	21.0 ± 0.1 *^c^*
11	p-Coumaric acid	39.9 ± 1.2 *^c^*	37.5 ± 0.6 *^c^*	84.8 ± 3.2 *^a^*	48.0 ± 1.4 *^b^*
12	Protocatechuic acid	29.1 ± 0.9 *^b^*	20.0 ± 0.6 *^c^*	32.2 ± 0.7 *^a^*	34.2 ± 0.7 *^a^*
13	Hyperoside	26.4 ± 0.5 *^a^*	4.64 ± 0.11 *^c^*	14.3 ± 0.4 *^b^*	14.9 ± 1.0 *^b^*
14	Luteolin	24.3 ± 1.3 *^a^*	7.10 ± 0.18 *^b^*	3.59 ± 0.02 *^c^*	-
15	Ferulic acid	21.7 ± 0.2 *^b^*	28.0 ± 0.4 *^a^*	15.8 ± 0.1 *^c^*	20.6 ± 1.0 *^b^*
16	Vanillin	8.36 ± 0.17 *^c^*	14.3 ± 3.2 *^bc^*	17.9 ± 1.4 *^ab^*	22.0 ± 1.1 *^a^*
17	(−)-Epicatechin	-	-	-	-
18	(+)-Catechin	-	-	-	-
19	2-Hydroxycinnamic acid	-	-	-	-
20	3,4-Dihydroxyphenylacetic acid	-	-	-	-
21	Eriodictyol	-	-	-	-
22	Gallic acid	-	-	-	-
23	Kaempferol	-	-	-	-
24	Pinoresinol	-	-	-	-
25	Quercetin	-	-	-	-
26	Resveratol	-	-	-	-
27	Sinapic acid	-	-	-	-
28	Syringic acid	-	-	-	-
29	Taxifolin	-	-	-	-

Values are expressed as mean ± standard deviation (n = 3). Values indicated by different superscript letters within the same row are statistically significantly different according to one-way ANOVA followed by Tukey’s HSD post hoc test (*p* < 0.05).

**Table 2:** Antioxidant activities of *O. sintenisii* extracts.

Assays	Flowers	Leaves	Stems	Roots	Trolox	EDTA
Phosphomolybdenum (EC_50_: mg/mL)	1.30 ± 0.03 *^bc^*	1.47 ± 0.002 *^cd^*	1.71 ± 0.11 *^d^*	1.03 ± 0.10 *^b^*	0.44 ± 0.04 *^a^*	-
CUPRAC reducing power (EC_50_: mg/mL)	2.72 ± 0.10 *^d^*	3.20 ± 0.01 *^e^*	2.39 ± 0.12 *^c^*	0.68 ± 0.02 *^b^*	0.16 ± 0.02 *^a^*	-
FRAP reducing power (EC_50_: mg/mL)	1.31 ± 0.06 *^d^*	1.77 ± 0.02 *^e^*	1.00 ± 0.06 *^c^*	0.29 ± 0.01 *^b^*	0.049 ± 0.003 *^a^*	-
DPPH radical (IC_50_: mg/mL)	12.41 ± 2.24 *^b^*	>20.00 *^c^*	9.94 ± 0.13 *^b^*	1.68 ± 0.05 *^a^*	0.24 ± 0.02 *^a^*	-
ABTS radical cation (IC_50_: mg/mL)	4.37 ± 0.39 *^cd^*	5.01 ± 0.07 *^d^*	3.83 ± 0.29 *^c^*	1.20 ± 0.02 *^b^*	0.17 ± 0.01 *^a^*	-
Ferrous ion chelating (IC_50_: mg/mL)	1.24 ± 0.02 *^b^*	1.15 ± 0.002 *^b^*	1.81 ± 0.09 *^c^*	1.68 ± 0.07 *^c^*	-	0.016 ± 0.005 *^a^*

Values are expressed as mean ± standard deviation (n = 3). Values indicated by different superscript letters within the same row are statistically significantly different according to one-way ANOVA followed by Tukey’s HSD post hoc test (*p* < 0.05). EDTAE mean.

**Table 3:** Enzyme inhibition activity of *O. sintenisii* extracts.

Samples	AChE (IC_50_: mg/mL)	BChE (IC_50_: mg/mL)	Tyrosinase (IC_50_: mg/mL)	α-Amylase (IC_50_: mg/mL)	α-Glucosidase (IC_50_: mg/mL)
Flowers	1.05 ± 0.003 ^e^	1.21 ± 0.02 ^d^	1.18 ± 0.002 ^c^	3.53 ± 0.06 ^b^	1.08 ± 0.001 ^bc^
Leaves	1.02 ± 0.003 ^d^	1.12 ± 0.01 ^c^	1.18 ± 0.001 ^c^	3.91 ± 0.01 ^c^	1.08 ± 0.02 ^bc^
Stems	1.00 ± 0.01 ^c^	1.07 ± 0.01 ^b^	1.18 ± 0.002 ^c^	4.84 ± 0.04 ^d^	1.03 ± 0.01 ^ab^
Roots	0.98 ± 0.003 ^b^	1.07 ± 0.004 ^b^	1.16 ± 0.01 ^b^	3.88 ± 0.01 ^c^	1.01 ± 0.001 ^a^
Galanthamine	0.0035 ± 0.0003 ^a^	0.0031 ± 0.0002 ^a^	-	-	-
Kojic acid	-	-	0.082 ± 0.002 ^a^	-	-
Acarbose	-	-	-	0.94 ± 0.04 ^a^	1.12 ± 0.03 ^c^

Values are expressed as mean ± standard deviation (n = 3). Values indicated by different superscript letters within the same row are statistically significantly different according to one-way ANOVA followed by Tukey’s HSD post hoc test (*p* < 0.05).

**Table 4:** Correlations among phenolic compounds and assays.

	TAP	DPPH	ABTS	CUPRAC	FRAP	FICA	AChEIA	BChEIA	TIA	AAIA	AGIA
DPPH	0.851										
ABTS	0.850	0.998									
CUPRAC	0.853	0.999	0.999								
FRAP	0.818	0.996	0.997	0.997							
FICA	−0.095	−0.540	−0.539	−0.533	−0.583						
RACI	0.975	0.919	0.917	0.920	0.895	−0.176					
AChEIA	0.332	0.743	0.753	0.750	0.767	−0.797					
BChEIA	0.005	0.478	0.486	0.484	0.509	−0.730	0.928				
TIA	0.790	0.951	0.950	0.951	0.954	−0.416	0.685	0.455			
AAIA	0.564	0.102	0.095	0.100	0.057	0.665	−0.555	−0.770	0.165		
AGIA	0.355	0.751	0.759	0.753	0.783	−0.901	0.916	0.810	0.652	−0.528	
Total flavonoid	−0.152	−0.615	−0.622	−0.616	−0.653	0.947	−0.936	−0.894	−0.515	0.708	−0.959
Total phenolic	0.850	0.998	0.998	0.998	0.998	−0.556	0.734	0.462	0.946	0.105	0.760
Rosmarinic acid	0.553	0.867	0.864	0.861	0.888	−0.879	0.831	0.630	0.749	−0.290	0.929
Chlorogenic acid	−0.278	−0.715	−0.724	−0.720	−0.747	0.864	−0.987	−0.929	−0.645	0.615	−0.957
Luteolin-7-O-glucoside	−0.380	−0.689	−0.706	−0.704	−0.701	0.452	−0.892	−0.856	−0.722	0.358	−0.711
Apigenin-7-O-glucoside	−0.059	−0.473	−0.492	−0.488	−0.501	0.583	−0.905	−0.970	−0.479	0.673	−0.738
Caffeic acid	−0.035	0.266	0.253	0.249	0.302	−0.861	0.395	0.330	0.088	−0.508	0.633
4-Hydroxybenzoic acid	−0.227	−0.368	−0.350	−0.347	−0.388	0.700	−0.221	−0.044	−0.191	0.134	−0.502
3-Hydroxybenzoic acid	−0.215	−0.349	−0.330	−0.328	−0.368	0.690	−0.205	−0.031	−0.167	0.135	−0.484
Verbascoside	0.819	0.968	0.961	0.961	0.970	−0.622	0.668	0.384	0.889	0.105	0.756
Apigenin	−0.095	−0.532	−0.549	−0.545	−0.563	0.685	−0.946	−0.983	−0.514	0.694	−0.818
Hesperidin	0.051	−0.131	−0.154	−0.154	−0.135	−0.056	−0.485	−0.626	−0.251	0.333	−0.194
p-Coumaric acid	−0.490	−0.064	−0.062	−0.069	−0.013	−0.787	0.459	0.603	−0.187	−0.916	0.570
Protocatechuic acid	0.381	0.643	0.627	0.625	0.666	−0.859	0.549	0.359	0.495	−0.242	0.749
Hyperoside	0.217	0.046	0.022	0.022	0.042	−0.109	−0.386	−0.581	−0.062	0.407	−0.100
Luteolin	−0.122	−0.534	−0.550	−0.547	−0.559	0.599	−0.924	−0.971	−0.539	0.639	−0.770
Ferulic acid	0.125	−0.214	−0.205	−0.199	−0.258	0.875	−0.408	−0.374	−0.057	0.582	−0.640
Vanillin	0.324	0.715	0.732	0.727	0.746	−0.706	0.939	0.894	0.704	−0.509	0.916

Data show the Pearson Correlation Coefficients between the parameters. TAP: total antioxidant activity by phosphomolybdenum method. AAIA, AGAI, AChEIA, BChEIA and TIA: α-amylase, α-glucosidase, acetylcholinesterase, butyrylcholinesterase and tyrosinase inhibition activities, respectively. ABTS and DPPH: ABTS and DPPH radical scavenging activities, respectively. CUPRAC and FRAP: CUPRAC and FRAP reducing power potential, respectively. FICA: Ferrous ion chelating activity. RACI: Relative antioxidant capacity index.

## Data Availability

The original contributions presented in this study are included in the article/Supplementary Material. Further inquiries can be directed to the corresponding author.

## Supplementary Materials

The following supporting information can be downloaded at: https://www.mdpi.com/article/10.3390/molecules31050840/s1, Table S1: ESI–MS/MS Parameters and analytical characteristics for the Analysis of Target Analytes by MRM Negative and Positive Ionization Mode; Table S2: Calibration curves and sensitivity properties of the method.
